# Combating Sarcopenia Through Nutrition: Anti-Inflammatory and Antioxidant Properties of *Aronia melanocarpa*

**DOI:** 10.3390/nu17213333

**Published:** 2025-10-23

**Authors:** Kalina Metodieva, Iliyan Dimitrov, Anelia Bivolarska

**Affiliations:** Department of Medical Biochemistry, Faculty of Pharmacy, Medical University of Plovdiv, 4002 Plovdiv, Bulgaria; iliyan.dimitrov@mu-plovdiv.bg (I.D.); anelia.bivolarska@mu-plovdiv.bg (A.B.)

**Keywords:** sarcopenia, oxidative stress, chronic inflammation, *Aronia melanocarpa*, polyphenols, functional foods

## Abstract

Introduction: Sarcopenia, the progressive age-related decline in skeletal muscle mass, strength, and function, represents a major contributor to morbidity, frailty, and reduced quality of life in older adults. Oxidative stress and chronic low-grade inflammation are increasingly recognized as central mechanisms driving its onset and progression, through pathways involving mitochondrial dysfunction, impaired satellite cell activity, and dysregulated protein turnover. Objective: The purpose of the following manuscript is to summarize current research on the molecular and cellular interactions between oxidative stress and inflammation in sarcopenia, as well as to assess *Aronia melanocarpa*’s potential as a nutritional intervention. Methods: A narrative review was conducted by searching PubMed, Scopus, and Web of Science for peer-reviewed literature published between 2000 and 2024. Keywords included “sarcopenia”, “oxidative stress”, “inflammation”, “*Aronia melanocarpa*”, “polyphenols”, and even “functional foods”. Eligible publications provided mechanistic, preclinical, or clinical findings on skeletal muscle biology and *A. melanocarpa* bioactivity. Results: This narrative review examines the relationship between oxidative stress and inflammation in sarcopenia, focusing on NF-κB-mediated inflammatory signaling, Nrf-2-dependent antioxidant defenses, myokines like myostatin and irisin, and macrophage polarization in muscle homeostasis. *Aronia melanocarpa* (black chokeberry) is highlighted as a polyphenol-rich fruit with a distinct profile of anthocyanins and proanthocyanidins that have strong antioxidant and anti-inflammatory properties. According to preclinical, clinical, and nutritional studies, *A. melanocarpa* bioactives modulate redox balance, suppress pro-inflammatory cytokine production, increase antioxidant enzyme activity, and regulate metabolic and regenerative signaling pathways important for skeletal muscle health. Conclusions: Overall, the data suggest *A. melanocarpa*’s potential as a functional food and nutraceutical candidate for the prevention and treatment of sarcopenia. However, further translational and clinical research is needed to determine the appropriate intake, bioavailability, and long-term efficacy in human populations.

## 1. Introduction

The aging process undergoes a dynamic and complex development, progressing over time. According to numerous studies, aging accelerates significantly after the fourth decade, ultimately leading to a terminal outcome. In essence, aging is a complex interaction of biological, physiological, and social elements, and its manifestation in every individual is different [1].

What is common among all, however, is the decline in organ function, which threatens the overall physiological state. This decline is closely linked to a reduced regenerative capacity of stem cells, which compromises tissue maintenance and repair [2]. At the cellular level, aging is associated with a permanent arrest of the cell cycle and cessation of mitosis [3]. In addition to irreversible cell cycle arrest, cellular senescence is characterized by profound alterations in chromatin structure, gene expression, organelle function, and overall cell morphology. Senescent cells change their secretory phenotype, transitioning to a senescence-associated secretory phenotype, which includes the secretion of proinflammatory cytokines [4]. This proinflammatory mediation alters the extracellular matrix, impairs stem cell function, and induces cellular transdifferentiation, which could spread the senescent phenotype to surrounding cells. In combination, these effects contribute to the development of systemic chronic low-grade inflammation, a hallmark of aging, also known as “inflammaging” [5]. In older individuals, this aseptic, chronic low-grade systemic inflammation is increasingly recognized as a common denominator of many age-related diseases [6].

Sarcopenia, as a skeletal muscle age-related disease, poses significant clinical, social, and economic challenges worldwide. The growing elderly population, particularly in both Western and rapidly developing countries, underscores the urgent need to elucidate the underlying mechanisms of sarcopenia to inform effective preventive and therapeutic strategies [7]. A molecular relationship between oxidative stress and inflammation has been hypothesized in the genesis of sarcopenia. Improved understanding of the immune mediators involved in skeletal muscle atrophy may provide valuable insights into the pathophysiological consequences of the diseases [8]. Bone marrow-derived immune cells, particularly infiltrating myeloid cells such as macrophages, neutrophils, and their progenitors, have a specific role in regulating cell proliferation, angiogenesis, and immune surveillance. A pathway of particular interest involves the generation and release of reactive oxygen species (ROS) by these myeloid cells under chronic inflammatory conditions [9]. The disrupted equilibrium between ROS production and antioxidant defense mechanisms in the elderly contributes to lipid peroxidation of cellular membranes and alterations in protein synthesis. These processes collectively result in diminished cellular proliferative capacity, increased susceptibility to mechanical injury, and a heightened inflammatory response, ultimately leading to compromised antioxidant defense and reduced regenerative potential of skeletal muscles in advancing age [10].

Robust evidence supports the critical role of nutrition in maintaining muscle mass, strength, and function among older adults, positioning nutritional interventions as key components in sarcopenia prevention and management [11]. For instance, *Aronia melanocarpa* extract promotes myogenic differentiation through protein kinase B (AKT) pathway activation, resulting in enhanced muscle mass and strength. It further upregulates both glycolytic and oxidative myofiber gene expression, regulating muscle-specific and metabolic pathways, suggesting its potential as a nutraceutical agent for mitigating muscle weakness and atrophy [12].

## 2. Materials and Methods

### 2.1. Methodology of Literature Review

This article presents a narrative review that synthesizes evidence from mechanistic, preclinical, and clinical studies examining the interplay between oxidative stress, chronic inflammation, and sarcopenia, with a particular focus on the antioxidant and anti-inflammatory potential of *Aronia melanocarpa*. A narrative review was chosen rather than a systematic or scoping review because the current body of literature is heterogeneous in study designs, outcomes, and models (ranging from cell-based assays and animal models to small clinical trials). This diversity makes it difficult to apply the strict requirements of systematic reviews, but a scoping review would be too wide and descriptive, not permitting in-depth exploration of mechanistic discoveries. The narrative format thus enables a focused integration of diverse findings and the identification of mechanistic themes and translational gaps that require further study.

#### 2.1.1. Databases and Search Strategy

A literature search was conducted in PubMed, Scopus, and Web of Science covering the period January 2000–December 2024. Search strategies combined relevant terms using Boolean operators. An example PubMed query was “sarcopenia” OR “skeletal muscle atrophy”; “oxidative stress” OR “reactive oxygen species”; “inflammation” OR “inflammaging” AND “NF-kB”; “*Aronia melanocarpa*” OR “black chokeberry” OR “polyphenols”. Equivalent adapted strings were applied to Scopus and Web of Science.

#### 2.1.2. Inclusion Criteria

Inclusion criteria were peer-reviewed studies in English reporting experimental, mechanistic, preclinical, or clinical findings relevant to skeletal muscle biology, oxidative stress, inflammation, or the bioactivity of *Aronia melanocarpa*.

#### 2.1.3. Exclusion Criteria

Exclusion criteria were commentaries, conference abstracts, publications not related to skeletal muscle or sarcopenia, and studies not addressing *Aronia melanocarpa* or its bioactive compounds.

#### 2.1.4. Filters

Searches were filtered by year of publication (2000–2024), language (English), and document type (meta-analysis, reviews, corrected and republished articles, original research articles, and clinical trials). Relevant articles were screened by title and abstract, followed by full-text assessment, and those meeting the eligibility criteria were synthesized narratively. This approach enabled the integration of diverse findings and highlighted current gaps that require further research.

#### 2.1.5. Strategies for Handling Conflicting Results

Since chronic inflammation is a key mechanism in sarcopenia, studies were included if they investigated oxidative stress or *Aronia melanocarpa* bioactivity, even when chronic inflammation was not the primary endpoint. The collected studies were organized into thematic categories, including mechanisms of sarcopenia and the roles of oxidative stress and inflammation; antioxidant and anti-inflammatory properties of *Aronia melanocarpa*, and potential interactions with muscle-specific regulatory factors such as myostatin and irisin. Within each theme, findings were qualitatively analyzed and narratively described, highlighting consistencies, discrepancies, and translational implications.

### 2.2. Application of AI in Manuscript Preparation

In the process of writing this publication, the creators utilized ChatGPT (OpenAI, version GPT-4 and GPT-5) to assist with the organization, formatting, and structural refinement of the text, as well as translation from Bulgarian into English, verification and correction of the manual translation, paraphrasing, refinement of language and academic phrasing, and reformatting the bibliography into the MDPI reference style. The writers carefully read, revised, and validated the manuscript, and they accept full responsibility for its final version. The scientific information was not created by artificial intelligence.

## 3. Results

### 3.1. Overview of Sarcopenia: Definition, Prevalence, and Clinical Relevance

Sarcopenia is an illness commonly defined by a gradual decrease in skeletal muscle mass and function, which is directly related to negative health outcomes [13]. As a consequence of an imbalance between protein synthesis and proteolysis, the ensuing muscle atrophy progressively compromises essential physical functions, including respiration, locomotion, and mobility [14]. Sarcopenia contributes significantly to the deterioration of quality of life and increases susceptibility to secondary conditions such as dyslipidemia, immunosuppression, metabolic syndrome, and cardiovascular disease [15,16,17]. A 2019 systematic review and meta-analysis, which included 41 studies and nearly 35,000 participants, found that sarcopenia affected about 11% of men and 9% of women. Prevalence rates were substantially higher among nursing home residents—around 51% for men and 31% for women—and among hospitalized older adults, where it reached roughly 23% for men and 24% for women [18].

The development of sarcopenia is influenced by multiple factors, including genetic predisposition, nutritional status, and comorbid clinical conditions [7]. Beyond primary sarcopenia, which is primarily age-related, secondary sarcopenia can arise due to various diseases such as diabetes mellitus and chronic obstructive pulmonary disease. Early identification and analysis of risk factors are critical for preventing the progression and complications associated with sarcopenia [19]. Factors such as physical inactivity, malnutrition, smoking, excessive sleep duration, diabetes, and other comorbidities have been implicated in increasing the incidence of sarcopenia [20,21]. Isometric muscle strength and power both decline significantly with advancing age; however, the annual loss of muscle power exceeds that of isometric strength. Consequently, muscle power has been proposed as a more sensitive indicator for the early detection of sarcopenia [22]. While sarcopenia is often associated with aging, secondary causes can induce sarcopenic changes in younger individuals. Contributory factors include metabolic syndrome, physical inactivity, poor nutrition, congenital and perinatal conditions, vitamin D deficiency, endocrine disorders, gut microbiota dysbiosis, neuromuscular diseases, organ failure, cancer, and other inflammatory states [23].

At the molecular level, several key factors are implicated in age-related sarcopenia. In elderly individuals, disruptions in mitochondrial function and biogenesis within skeletal muscle significantly contribute to declines in muscle mass and performance [24,25]. Aging increases skeletal muscle susceptibility to oxidative stress, particularly during periods of rest and disuse atrophy, underscoring oxidative stress as a central driver of muscle loss in these contexts. Chronic oxidative stress also plays a pivotal role in the pathogenesis of diseases that promote muscle wasting [26]. However, mitochondrial dysfunction and increased oxidative stress are the central driving forces for age-related skeletal muscle abnormalities. Excess oxidative stress in aged muscle leads to excitation–contraction uncoupling, disrupted calcium homeostasis, apoptosis-mediated fiber loss, atrophy of residual fibers, satellite cell dysfunction, and impaired muscle regeneration—all contributing to reduced muscle mass, strength, and function [27]. The primary enzymatic antioxidant defenses counteracting oxidative stress include superoxide dismutase (SOD), catalase (CAT), and glutathione peroxidase (GPx). The activities of these enzymes are modulated by factors such as physical exercise, nutritional status, and the aging process. Furthermore, dietary and endogenous antioxidants play a critical role in maintaining the redox homeostasis of muscle cells, thereby mitigating ROS-induced intracellular damage and preserving muscle function [28].

ROS generation occurs in multiple cellular compartments, including the cytoplasm, plasma membrane, endoplasmic reticulum, mitochondria, and peroxisomes, as well as via enzymes such as NADPH (nicotinamide adenine dinucleotide phosphate) oxidase. The effects of ROS vary depending on their origin and cellular localization, exerting both physiological and pathological functions [29]. The remarkable interplay between oxidative stress and inflammation reflects how ROS regulate the transcription of nuclear factor kappa B (NF-κB)-dependent genes, while ROS themselves modulate NF-κB activity. NF-κB transcription factors, in turn, play a pivotal role in coordinating inflammatory responses and maintaining immune function, thereby underscoring the bidirectional association of oxidative stress and inflammation [30].

Chronic inflammation in skeletal muscle impairs regenerative capacity, affecting satellite cell activation, and promotes accumulation of fibro-adipogenic progenitors and extracellular matrix deposition, impeding effective muscle repair and differentiation [31]. Satellite cell function is impaired by increased oxidative stress in aging muscle. Alongside changes in systemic factors, many of which remain largely unknown, this leads to compromised muscle regeneration and accelerates the progression of sarcopenia [32].

The antioxidant and anti-inflammatory molecules contained in *Aronia melanocarpa* may influence these pathophysiological processes by limiting oxidative stress and modulating the immune response in older individuals [33].

Mass-spectrometry-based proteomic analyses of aging human and animal skeletal muscle reveal a consistent trend: a fiber-type shift from fast-twitch (Type II) to slow-twitch (Type I) fibers during aging, most likely due to the preferential susceptibility of fast-twitch fibers to atrophy (Table 1). These changes are reflected in the differential expression of contractile proteins, including various myosin heavy and light chains, actin, troponin, and tropomyosin, which serve as biomarkers for fiber-type transitions in sarcopenic muscles [34]. Since fast type II fibers are particularly susceptible to atrophy, the polyphenols from *A. melanocarpa* may exert a protective effect by enhancing mitochondrial function and reducing inflammation-induced damage in these fibers [12].

### 3.2. Aronia melanocarpa: A Nutritional Powerhouse for Reducing Oxidative Stress and Inflammation

Functional foods are specially designed to include bioactive compounds or beneficial microorganisms at concentrations that are both safe and effective, aiming to support health maintenance and reduce disease risk. These formulations often incorporate nutrients, dietary fibers, phytochemicals, or probiotics, which contribute to their physiological benefits beyond basic nutrition [35]. Fruits and vegetables contribute essential micronutrients to the diet, including a wide range of vitamins and minerals, and serve as rich sources of phytochemicals that exert antioxidant, estrogenic, anti-inflammatory, and other protective biological effects [36].

*Aronia melanocarpa*, commonly known as black chokeberry, is an underutilized fruit species increasingly recognized for its high nutritional value and potent antioxidant properties, particularly due to its rich polyphenol content [37]. Native to eastern North America, this deciduous shrub was traditionally employed by Indigenous peoples such as the Abnaki and Potawatomi tribes for therapeutic purposes, including cold treatments. In recent years, *A. melanocarpa* has gained popularity in Eastern European countries and Russia, where it is cultivated for the production of various food products, including juices, jams, teas, wines, and natural colorants [38].

Extracts from *Aronia* have been demonstrated to possess geroprotective properties, as potential mechanisms of action involve hormesis, activation of antioxidant defenses, and anti-inflammatory activity [39].

The chemical composition of black chokeberries has been extensively studied, particularly concerning the bioactive compounds that contribute to their health benefits. Phytochemical profiling has demonstrated that *A. melanocarpa* is an abundant source of phenolic compounds, including procyanidins, anthocyanins, phenolic acids, and their derivatives [40]. Among the most significant nutritional constituents are polyphenols, carbohydrates, essential minerals, and vitamins, all of which contribute to its documented physiological and health-promoting effects. A growing body of in vitro and in vivo research has confirmed the wide-ranging biological activities of these compounds, which include antioxidant [41], anti-inflammatory [42], cardioprotective [43], antiviral [44], anticancer [45], antiplatelet [46], and antidiabetic properties [47].

Polyphenols in *Aronia* can be broadly classified into two main categories: flavonoids and phenolic acids. Flavonoids exist in plants either as free aglycones or as glycosidic conjugates, including O- and *C*-glycosides [48]. The major flavonoid subgroups present in *Aronia* include anthocyanins, flavonoids, and flavanols, while the phenolic acid profile is dominated by chlorogenic acid and its isomers. Among these, proanthocyanidins have been identified as the principal contributors to the fruit’s antioxidant capacity. The predominant anthocyanins are cyanidin derivatives, particularly cyanidin-3-O-arabinoside and cyanidin-3-O-galactoside, with cyanidin-3-O-galactoside being recognized as a key phenolic compound associated with elevated antioxidant and radical-scavenging activity [49].

Flavonoids exert protective effects by neutralizing ROS produced under environmental or physiological stress conditions. They also modulate the activity of key ROS-generating enzymes, such as peroxidase, lipoxygenase, and xanthine oxidase [48]. A polyphenol-rich diet has thus been shown to mitigate chronic disease risk by regulating various physiological pathways, including redox homeostasis, enzymatic activity, cell proliferation, and intracellular signaling mechanisms. Despite their demonstrated bioactivity, polyphenols generally exhibit low oral bioavailability. This is primarily due to extensive biotransformation via phase I and phase II metabolic reactions occurring in enterocytes, hepatocytes, and through microbial metabolism in the gut [50]. For example, the bioavailability of the predominant anthocyanins and proanthocyanidins found in *Aronia* is estimated to be below 6% of the ingested dose [51]. Despite their limited bioavailability, polyphenols from *Aronia* remain highly biologically active, presenting what is often referred to as the “low bioavailability/high bioactivity paradox” [52]. Although this paradox remains to be fully elucidated, several studies have reported that polyphenols derived from *Aronia* exert their biological effects in a dose-dependent manner, with lower doses producing more favorable outcomes than higher ones [53].

Black chokeberry anthocyanins are being actively investigated for their anticancer potential, owing to their capacity to modulate oxidative stress and inflammatory pathways [52]. For instance, *Aronia* concentrate has been shown to protect Raw 264.7 macrophage cells from NF-κB pathway activation while suppressing the release of key pro-inflammatory molecules such as tumor necrosis factor-alpha (TNF-α), interleukin 6 (IL-6), and interleukin 8 (IL-8) from human peripheral monocytes. When combined with sodium selenite, it demonstrates a synergistic effect in inhibiting prostaglandin E2 (PGE-2) production, cytokine secretion, and NF-κB signaling [54]. In addition, *Aronia* extract has been shown to suppress ROS formation in macrophages, leading to decreased NF-κB signaling and subsequent inhibition of iNOS-mediated nitric oxide (NO) synthesis [55].

Complementary findings reveal in more detail that black chokeberry polyphenols (BCPs) significantly attenuate nitric oxide (NO) production and reduce the expression of pro-inflammatory mediators, including monocyte chemoattractant protein 1 (MCP-1), interleukin 1β (IL-1β), TNF-α, and IL-6. This anti-inflammatory action is paralleled by a dose-dependent modulation of oxidative stress biomarkers such as GPx, CAT, and malondialdehyde (MDA), ultimately contributing to improved glucose tolerance, reduced systemic inflammation, and mitigation of high-fat diet-induced obesity in animal models [56].

**Figure 1:**
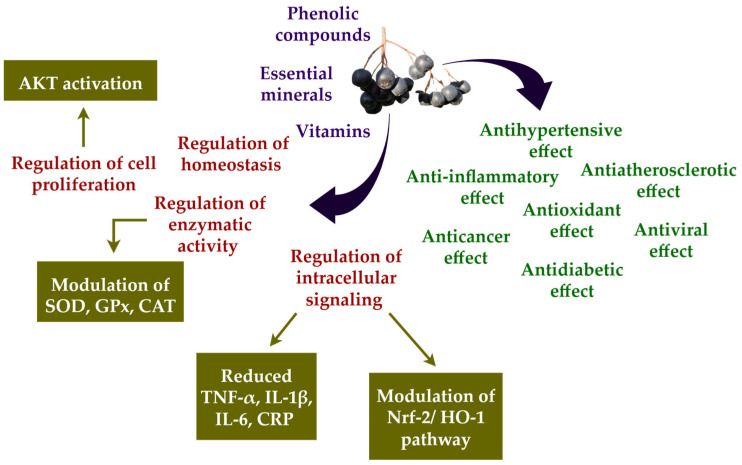
Proposed mechanisms underlying the biological effects of black chokeberry (*Aronia melanocarpa*). The fruit is rich in phenolic compounds, vitamins, and essential minerals, which contribute to the regulation of homeostasis, enzymatic activity, and intracellular signaling. These actions involve AKT activation and modulation of key antioxidant enzymes, such as SOD, GPx, and CAT. As a result, pro-inflammatory mediators, including TNF-α, IL-1β, IL-6, and CRP, are reduced, while the Nrf-2/HO-1 signaling pathway is activated. Collectively, these molecular effects translate into a wide range of physiological benefits, including antihypertensive, anti-inflammatory, anti-atherosclerotic, antioxidant, anticancer, antiviral, and antidiabetic properties. Abbreviations: AKT, protein kinase B; CAT, catalase; CRP, *C*-reactive protein; GPx, glutathione peroxidase; HO-1, heme oxygenase 1; IL-1β, interleukin 1β; IL-6, interleukin 6; Nrf-2, nuclear factor erythroid 2-related factor 2; SOD, superoxide dismutase; TNF-α, tumor necrosis factor α.

In addition to its rich polyphenolic content, the polysaccharide fraction of *Aronia melanocarpa* (AMP) has demonstrated notable neuroprotective effects by modulating key signaling pathways such as AMP-activated protein kinase (AMPK)/Sirtuin 1 (SIRT 1)/NF-κB and nuclear factor erythroid 2-related factor 2 (Nrf-2)/heme oxygenase 1 (HO-1), which contribute to the reduction of inflammation and oxidative stress in the aging brain. Furthermore, AMP activates the phosphoinositide 3-kinase (PI3K)/mammalian target of rapamycin) mTOR/AKT pathway and regulates apoptosis-related protein families, suggesting a promising role in preventing age-associated neurological decline [57]. Complementing these findings, recent evidence shows that supplementation with *Aronia* also positively influences systemic inflammatory biomarkers by significantly reducing *C*-reactive protein (CRP), TNF-α, and IL-6 levels while elevating the anti-inflammatory cytokine interleukin 10 (IL-10). This is accompanied by enhanced antioxidant defense mechanisms, as indicated by increased activities of enzymes such as SOD, GPx, and CAT (Figure 1), further supporting its potential in mitigating oxidative stress and inflammation [33]. Taken together, these findings underscore the significant potential of *A. melanocarpa* as a functional food with multifaceted health benefits, particularly in modulating oxidative stress and inflammation, thereby supporting its therapeutic application in age-related and chronic inflammatory conditions [58].

### 3.3. The Role of Irisin and Myostatin in Overall Muscle State

In recent years, it has been increasingly recognized that muscle contraction triggers the release of peptides and proteins termed myokines, conferring secretory properties to skeletal muscle tissue. Myokines function as important biological mediators with autocrine, paracrine, or endocrine effects, influencing essential processes such as metabolism, angiogenesis, and inflammation at a systemic level [59].

One well-studied myokine is myostatin (MSTN), a pro-oxidant that stimulates ROS production in muscle cells. Myostatin, also known as growth differentiation factor 8 (GDF-8) and a member of the transforming growth factor-beta (TGF-β) superfamily, contributes to muscle atrophy by inducing protein degradation [60]. It causes oxidative stress in skeletal muscle cells through activation of TNF-α signaling mediated by NF-κB and NADPH oxidase. Elevated TNF-α levels, in turn, enhance myostatin expression, creating a feedback loop that exacerbates muscle wasting by activating intracellular proteolytic pathways, notably the ubiquitin–proteasome system. Therefore, inhibiting myostatin-induced ROS production represents a promising strategy to minimize muscle loss characteristic of sarcopenia [61].

**Figure 2:**
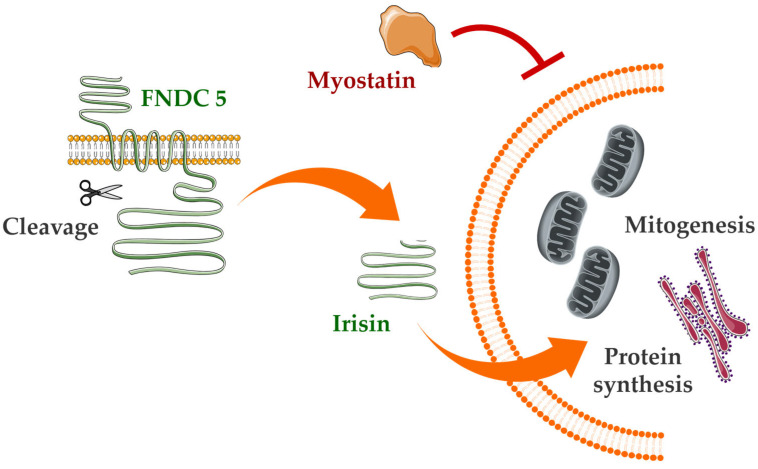
Mechanism of FNDC-5 cleavage and subsequent irisin-mediated effects on muscle cells. FNDC-5, a transmembrane protein, undergoes proteolytic cleavage to release the myokine irisin. Irisin then acts on muscle cells to promote mitochondrial biogenesis (mitogenesis) and enhance protein synthesis, contributing to improved muscle function and metabolic regulation. Myostatin, a negative regulator of muscle growth, inhibits this pathway, counteracting irisin’s anabolic effects. This schematic highlights the interplay between FNDC-5-derived irisin signaling and myostatin-mediated inhibition in skeletal muscle physiology. Abbreviations: FNDC-5, fibronectin type III domain-containing protein 5.

Another intriguing myokine is irisin, a cleaved form of fibronectin type III domain-containing protein 5 (FNDC5), produced during physical exercise and shown to have multiple health benefits (Figure 2). Both irisin and its precursor FNDC5 are downregulated in conditions of reduced protein synthesis within muscle [62]. Phenotypic studies in mice demonstrate that FNDC5/irisin deficiency in aging individuals leads to aggravated muscle atrophy, including decreased grip strength and reduced muscle mass. Conversely, administration of recombinant irisin intraperitoneally in aged or senescent mice improves sarcopenic symptoms and attenuates age-related adipose tissue expansion [63]. Irisin is also known to modulate glucose metabolism, inflammation, and the immune response, exhibiting notable anti-inflammatory and anti-apoptotic effects. It reduces lipopolysaccharide (LPS)-induced liver injury and inflammatory cytokine production. In vitro, LPS-treated Raw 264.7 macrophage-like cells of animal models (cell line derived from a mouse tumor induced by Abelson murine leukemia virus) show elevated NLRP3 (NOD-, LRR- and pyrin domain-containing protein 3) inflammasome activity, inflammation, and ROS- and NF-κB-mediated apoptosis—effects that can be reversed by irisin treatment [64]. Interestingly, extracts from *A. melanocarpa* demonstrate the ability to activate the AKT signaling pathway, which is directly related to myogenesis and may potentiate the effects of myokines such as irisin while simultaneously reducing the negative impact of myostatin [12].

### 3.4. Role of Oxidative Stress and Inflammation in the Development of Sarcopenia

Oxidative stress is a fundamental contributor to cellular aging in general. The uninterrupted progression of regenerative processes is essential for maintaining proper tissue homeostasis and functional integrity [65].

Living organisms are continuously exposed to oxidative stress, which induces oxidative modifications in deoxyribonucleic acid (DNA). One such modification is 8-hydroxy-2′-deoxyguanosine (8-OHdG), a well-established biomarker of oxidative stress and DNA damage detectable both in physiological fluids and cells [66]. Another sign of oxidative stress is MDA, a result of polyunsaturated fatty acid peroxidation within cells. Increased free radical production results in elevated MDA levels [67].

Age-related conditions such as sarcopenia are directly linked to increased oxidative stress in cells [68]. During aging, basal ROS levels rise in muscle fibers and satellite cells, causing oxidation of DNA, proteins, and lipids, disrupting myogenic protein metabolism, and inhibiting skeletal muscle cell differentiation. Specifically, elevated ROS such as hydrogen peroxide (H_2_O_2_) can suppress phosphorylation of mTOR, AKT, and other downstream signaling molecules, leading to muscle atrophy or sarcopenia due to inhibited protein synthesis [69]. Thus, the pathogenesis of geriatric diseases, including sarcopenia, arises from an imbalance between antioxidant defenses and ROS production, with antioxidant capacity reduced and ROS generation unchanged or increased [70].

The cellular response to ROS production is crucial for preventing further oxidative damage and maintaining cell survival. ROS levels are directly associated with NF-κB-dependent genes, while NF-κB activity is, in turn, modulated by ROS. ROS induce the expression of pro-inflammatory mediators such as IL-1β, TNF-α, interleukin 4 (IL-4), interleukin 12 (IL-12), and IL-6 through NF-κB signaling. NF-κB proteins are transcription factors that play a central role in inflammation and immunity, highlighting the interplay between oxidative stress and inflammation [71,72]. Aerobic cells express varying levels of three key antioxidant enzymes: SOD, CAT, and GPx. These enzymes are essential for cell survival, as their inhibition leads to cell cycle arrest and cell death [73]. Cells also possess transcription factors involved in protective mechanisms. Oxidative stress, defined as an imbalance between ROS production and elimination by defense systems, can lead to chronic inflammation [74]. One such transcription factor is Nrf-2, which supports cellular defenses against oxidative stress and inflammation by inducing the expression of antioxidant genes, including HO-1 and SOD. Nrf-2 deficiency correlates with increased oxidative stress, which promotes NF-κB activity and results in elevated production of pro-inflammatory mediators [75]. Under basal conditions, Nrf2 remains sequestered in the cytoplasm through its association with Keap 1 (Kelch-like ECH associating protein 1), which facilitates its ubiquitin-mediated degradation. However, upon exposure to ROS or electrophilic insults, Nrf2 dissociates from Keap 1 and is stabilized, allowing its translocation into the nucleus where it activates the transcription of antioxidant and cytoprotective genes [76]. Notably, Nrf-2 expression declines with age, leading to dysregulated antioxidant defenses and increased cellular oxidative stress [77].

Evidence suggests that polyphenol-based therapies not only prevent inflammation but also inhibit free radical formation. Furthermore, they activate multiple protective genes such as Nrf-2, AMPK, SOD, CAT, HO-1, SIRT 1, and other key proteins involved in mitochondrial biogenesis [78].

The polyphenols in *A. melanocarpa* have been shown to activate Nrf2-dependent antioxidant defense and suppress NF-κB-mediated inflammation—two key mechanisms in the pathogenesis of sarcopenia [51,79].

### 3.5. Macrophages as Part of the Balance Between Pro- and Anti-Inflammatory Responses

Macrophages represent a highly heterogeneous and multifunctional group of differentiated mononuclear phagocytes. They are involved in all phases of the immune response, from its initiation, through the development and regulation of the inflammatory process, to the complete restoration of tissues. Macrophages also participate in tissue development, remodeling, and the maintenance of tissue homeostasis [80]. Classically activated macrophages, or M1 macrophages, exhibit a pro-inflammatory phenotype, secreting cytokines such as IL-1β, IL-6, and TNF-α. M1 macrophages are characterized by high expression levels of surface markers, including cluster of differentiation 80 (CD80), cluster of differentiation 86 (CD86), and major histocompatibility complex molecules (MHC class II), relying predominantly on anaerobic glycolysis to meet their energy demands while producing substantial amounts of ROS [81]. Alternatively activated (non-classical) macrophages, or M2 macrophages, are associated with tissue repair and restoration of tissue homeostasis. They secrete anti-inflammatory molecules, including IL-10 and TGF-β, as well as extracellular matrix proteins that support tissue healing. M2 macrophages also express numerous surface markers, such as cluster of differentiation 163 (CD163) and cluster of differentiation 206 (CD206). However, they produce lower levels of ROS, relying primarily on β-oxidation and oxidative phosphorylation to fulfill their energy requirements [82].

The ratio between the two macrophage phenotypes, M1 and M2, can fluctuate over the course of the inflammatory response and depends on microenvironmental factors [83]. An altered balance between M1 and M2 macrophages is considered potentially detrimental, as both prolonged M1 activation and impaired M2 functions may trigger and perpetuate chronic inflammation [84].

It has been observed that the healing process in the musculoskeletal system of elderly individuals is delayed, a phenomenon closely linked to dysregulated inflammatory control [85]. The maintenance of a pro-inflammatory microenvironment during aging leads to the activation of adaptive compensatory mechanisms, which in turn result in delayed tissue recovery [86].

Data from experimental models suggest that biologically active compounds in *A. melanocarpa* may help support the balance between M1 and M2 macrophages, thereby facilitating skeletal muscle regeneration [87].

## 4. Limitations

Despite the promising findings for *Aronia melanocarpa*’s antioxidant and anti-inflammatory benefits in the context of sarcopenia, some significant limitations must be recognized. First, the majority of current research is based on in vitro and animal models, which may not fully translate to human physiology. Existing clinical trials are limited in number, with small sample sizes, brief intervention periods, and varied groups, limiting the generalizability of findings. Furthermore, several studies use a variety of Aronia-derived products (juices, extracts, powders), making dosage and formulation standardization challenging. Another major issue is the low oral bioavailability of polyphenols, which raises questions about the effective doses required to exert biological effects in skeletal muscle.

## 5. Conclusions

Sarcopenia is a complex, multifactorial condition in which oxidative stress and chronic inflammation play central roles. These processes accelerate mitochondrial dysfunction, impair anabolic signaling, and limit muscle regenerative capacity, ultimately leading to a decline in functional capacity. Nutritional strategies that target these pathways are therefore of high interest.

*Aronia melanocarpa* is among the richest dietary sources of polyphenols, with compelling preclinical evidence supporting its ability to activate antioxidant defenses, suppress pro-inflammatory signaling, and enhance muscle metabolism through AKT and mTOR pathways. These mechanisms suggest a plausible role for *Aronia* in protecting vulnerable type II fibers and mitigating age-related muscle atrophy.

Nonetheless, clinical data to date are limited, short-term, and heterogeneous, with small sample sizes and a variety of formulations. Polyphenols have low absorption, which hampers translation into effective therapies. Well-controlled, long-term clinical trials using standardized *Aronia* formulations are required to assess whether the intriguing molecular and preclinical findings transfer into considerable increases in muscle mass, strength, and function in older individuals.

Overall, *Aronia melanocarpa* has promising but preliminary prospects as a safe and accessible dietary component that may help maintain muscular health during aging. Its potential as a nutraceutical candidate should be viewed as speculative and exploratory until more substantial clinical data are obtained.

## Figures and Tables

**Table 1:** Features of skeletal muscle fiber types. The following table outlines the key structural and functional properties of the three major skeletal muscle fiber types: Type I, Type IIa, and Type IIx/IIb. Type I fibers, also known as slow-twitch fibers, have the slowest contraction speed, a high mitochondrial density, low glycogen content, red pigmentation, and excellent fatigue resistance, making them ideal for endurance and continuous activity. Type IIa fibers exhibit an intermediate phenotype, with moderate contraction speed, fewer mitochondria than Type I, high glycogen content, red color, and moderate fatigue resistance, allowing for a balance of power production and endurance. Type IIx/IIb, also known as fast-twitch fibers, contract the fastest, have the fewest mitochondria, a high glycogen content, white pigmentation, and low fatigue resistance, making them ideal for rapid, high-intensity, short-duration movements. Collectively, these characteristics illustrate the metabolic and functional diversity of skeletal muscle fibers, which underlies their distinct contributions to muscle performance and adaptation.

Features	Type I Fibers	Type IIa Fibers	Type IIb/IIx Fibers
Speed of contraction	Slowest	Intermediate	Fastest
Mitochondria	Many	Fewer	Least
Glycogen content	Low	High	High
Color	Red	Red	White
Fatigue resistance	High	Moderate	Low
Activity duration	Long	Moderate	Short

## Data Availability

The original contributions presented in this study are included in the article. Further inquiries can be directed to the corresponding author.

## Abbreviations

The following abbreviations are used in this manuscript:

AKT	Protein kinase B
AMPK	Adenosine monophosphate-activated protein kinase
CAT	Catalase
CD 80	Cluster of differentiation 80
CD 86	Cluster of differentiation 86
CD 163	Cluster of differentiation 163
CD 206	Cluster of differentiation 206
CRP	C-reactive protein
DNA	Deoxyribonucleic acid
FNDC-5	Fibronectin type III domain-containing protein 5
GDF-8	Growth differentiation factor 8
GPx	Glutathione peroxidase
HO-1	Heme oxygenase 1
IL-1β	Interleukin 1β
IL-4	Interleukin 4
IL-6	Interleukin 6
IL-8	Interleukin 8
IL-10	Interleukin 10
IL-12	Interleukin 12
Keap 1	Kelch-like ECH-associating protein 1
LPS	Lipopolysaccharide
MCP-1	Monocyte chemoattractant protein 1
MDA	Malondialdehyde
MHC II	Histocompatibility complex molecules class II
MSTN	Myostatin
mTOR	Mammalian target of rapamycin
NADPH	Nicotinamide adenine dinucleotide phosphate (reduced form)
NF-κB	Nuclear factor kappa B
NLRP-3	NOD-, LRP-, and pyrin domain-containing protein 3
NO	Nitric oxide
Nrf-2	Nuclear factor erythroid 2-related factor 2
8-OHdG	8-hydroxy-2᾿-deoxyguanosine
PGE-2	Prostaglandin E2
PI3K	Phosphoinositide 3-kinase
Raw 264.7	Cell line derived from a mouse tumor induced by Abelson murine leukemia virus
ROS	Reactive oxygen species
SIRT 1	Sirtuin 1
SOD	Superoxide dismutase
TGF-β	Transforming growth factor-beta
TNF-α	Tumor necrosis factor-alpha
